# Conditioning effect of transcranial magnetic stimulation evoking motor‐evoked potential on V‐wave response

**DOI:** 10.14814/phy2.12191

**Published:** 2014-12-11

**Authors:** Sidney Grosprêtre, Alain Martin

**Affiliations:** 1 Faculté des sciences du sport, INSERM U1093, Université de Bourgogne, Dijon, France

**Keywords:** antidromic collision, flexor carpi radialis, transcranial magnetic stimulation, triceps surae

## Abstract

The aim of this study was to examine the collision responsible for the volitional V‐wave evoked by supramaximal electrical stimulation of the motor nerve during voluntary contraction. V‐wave was conditioned by transcranial magnetic stimulation (TMS) over the motor cortex at several inter‐stimuli intervals (ISI) during weak voluntary plantar flexions (*n* = 10) and at rest for flexor carpi radialis muscle (FCR;* n* = 6). Conditioning stimulations were induced by TMS with intensity eliciting maximal motor‐evoked potential (MEP_max_). ISIs used were ranging from −20 to +20 msec depending on muscles tested. The results showed that, for triceps surae muscles, conditioning TMS increased the V‐wave amplitude (~ +250%) and the associated mechanical response (~ +30%) during weak voluntary plantar flexion (10% of the maximal voluntary contraction ‐MVC) for ISIs ranging from +6 to +18 msec. Similar effect was observed at rest for the FCR with ISI ranging from +6 to +12 msec. When the level of force was increased from 10 to 50% MVC or the conditioning TMS intensity was reduced to elicit responses of 50% of MEP_max_, a significant decrease in the conditioned V‐wave amplitude was observed for the triceps surae muscles, linearly correlated to the changes in MEP amplitude. The slope of this correlation, as well as the electro‐mechanical efficiency, was closed to the identity line, indicating that V‐wave impact at muscle level seems to be similar to the impact of cortical stimulation. All these results suggest that change in V‐wave amplitude is a great index to reflect changes in cortical neural drive addressed to spinal motoneurons.

## Abstract (precis)

This study aimed to condition V‐wave by transcranial magnetic stimulation (TMS), allowing assessing the amplitude and time‐delays of the descending drive. Thus, by modulating TMS intensities, levels of voluntary contraction and inter‐stimuli intervals, we were able to estimate the possible site of the collision allowing recording of V‐wave and the link with motor‐evoked potential magnitude and V‐wave amplitude. These results bring new knowledge about the modulation of the V‐wave and its interpretation.

## Introduction

Initially described by Upton et al., in 1971, the volitional wave response (V‐wave), an electrophysiological variant of the Hoffman (H) reflex, has been widely used as a response that could provide information regarding the magnitude of efferent neural drive addressed to spinal alpha‐motorneurons (Mn*α*). This wave, evoked by supramaximal electrical nerve stimulation delivered during a voluntary contraction, consists of afferent volley impulses that reach the muscle thanks to the collision of the descending neural drive with the antidromic volleys generated in motor axons (Upton et al. 1971). The size of this response, while being sensitive to the ongoing neural output addressed to Mnα (Aagaard et al. 2002; Duclay and Martin 2005; El Bouse et al. 2013), is also affected by Mnα responsiveness and synaptic transmission efficacy between Ia afferent and Mnα including pre‐and post‐synaptic mechanisms (Grosprêtre and Martin 2012; Racinais et al. 2013).

Because V‐wave is a spinal reflex that partly involves the same neural circuitry than the H‐reflex (Upton et al. 1971), several studies have attempted to analyze, by recording both responses, the potential mechanisms mediating neural adjustment following training (Aagaard et al. 2000; Duclay et al. 2008; Vila‐Chã et al. 2012), fatiguing exercise (Racinais et al. 2007; Girard et al. 2011) or limb suspension (Seynnes et al. 2010). For example, increased V‐wave amplitude without changes in H‐reflex size has been already observed following strength training (Vila‐Chã et al. 2012) suggesting that collision between antidromic volleys and descending drive allows a higher number of motor axons to be available for afferent volley discharge. This greater collision is possible due to an increased descending neural drive addressed to the motoneuronal pool indicating that neural adaptations occur partly at supraspinal level (Vila‐Chã et al. 2012).

However, the different stimulus intensities used to evoke the H‐reflex and the V‐wave could activate distinct populations of afferents and motoneurons that do not allow ruling out contribution of spinal mechanisms (Gondin et al. 2006). Most of the studies reported lower V‐wave size in comparison to H‐reflex amplitude (Aagaard et al. 2002; Duclay et al. 2008) and others did not (Seynnes et al. 2010; Vila‐Chã et al. 2012). Despite the fact that discrepancies could be partly due to differences in how the reflexes are elicited, these results raise the question about the nature of collisions between descending neural drive and antidromic volleys generated by supramaximal stimulation of the motor nerve (Grosprêtre and Martin 2012; Racinais et al. 2013).

To investigate the collision between the antidromic volley and the descending drive, the conditioning effect of transcranial magnetic stimulation (TMS) on maximal M wave (M_max_), induced by supramaximal electrical stimulation of motor nerve, could be examined. The non‐invasive technique of TMS is used to study descending corticospinal inputs addressed to spinal motoneurons (Rothwell et al. 1991). Despite the fact that such single stimulation evokes corticospinal volleys consisting of multiple waves, it has been shown that it produces similar recruitment order and rate coding of motoneurons to that found for voluntary contraction (Bawa and Lemon 1993). Also, considering MEP and V‐wave latencies, analysis of the conditioning effect of TMS on M_max_ by varying inter‐stimuli intervals (ISI) should help to define the segmental part of the neural drive where the collision between corticospinal and antidromic volleys generated in motor axons takes place. Moreover, studying the electrophysiological and mechanical responses of such conditioning maneuver applied during various submaximal voluntary contraction should allow to investigate the population of motor units involved in the V‐wave response.

The aim of the present study was to investigate the effects of conditioning TMS on V‐wave amplitude at various submaximal voluntary contractions and conditioning intensities. Electrophysiological and mechanical responses analysis of conditioning and non‐conditioning stimulations could give valuable insights on the physiological meaning of V‐wave. We can hypothesis that conditioned V‐wave amplitude will follow the intensity and magnitude of conditioning TMS used. The analyze of the effective inter‐stimulii intervals, according to the latencies of the several responses, should demonstrate that the collision between cortico‐spinal volley and peripheral motoneurons activation should occur between stimulation site and motoneuronal pool.

## Material and Methods

Experiments were performed on ten young healthy subjects (age: 24.20 ± 1.9 years, height: 176.7 ± 22 cm, weight: 70.33 ± 2.79 kg). None of them reported neurological or physical disorders. They were all volunteers and, after being fully informed about the investigation and possible related risks, gave informed consent to participate in the study. All experimental procedures were performed in accordance with the Declaration of Helsinki and approved by the Regional Ethics Committee.

Three experiments were carried out during sessions of about 3 h separated by at least 3 days. Two experiments were designed on triceps surae muscles (*n* = 10) and the third on flexor carpi radialis (FCR; *n* = 6).

### Mechanical recording

All experiments were performed in a sitting position using an isokinetic dynamometer (Biodex Shirley, NY). For plantar flexor muscles, measurements were recorded on the right leg with hip and knee joints at 90° (0°= full extension) and ankle joint at 90° (i.e., angle between the leg and the sole of the foot). The ankle was firmly strapped to the dynamometer with the motor axis aligned with the external malleolus of the ankle. For FCR muscle experiment, the right arm of the subject was aligned with the trunk (shoulder angle set at 0°) and the elbow joint was flexed at 90° (0°= full extension). The dynamometer's motor axis was aligned with the styloid process of the ulna. Subjects' hand was firmly strapped in neutral position. During all experiments, particular care was taken in monitoring subjects' posture and avoiding head rotations to maintain constant cortico‐vestibular influences on spinal excitability (Schieppati 1987). Trunk was stabilized by two crossover shoulder harnesses and head rotations and tilt were avoided by a collar fastened to the headrest of the seat.

To assess maximal plantar and wrist flexion torque, subjects were asked to perform two maximal voluntary contractions (MVC). For submaximal contraction, they were instructed to reach the level of force, monitored on a screen, then to hold it for 4 sec. The mechanical signals were digitized on‐line (sampling frequency 5 kHz) and stored for analysis in TIDA software (Heka Elektonik, Lambrecht/Pfalz, Germany).

### Myoelectrical activity

EMG activity was recorded from four muscles of the right leg (the *tibialis anterior,* TA; the *soleus*, SOL; the *gastrocnemius medialis*, MG and *lateralis*, LG) and one muscle of the right forearm (FCR). After shaving and dry‐cleaning the skin with alcohol to keep low impedance (<5kΩ), the EMG signals were obtained by using two silver‐chloride surface electrodes (8 mm diameter) placed at an inter‐electrode center‐to‐center distance of 2 cm. For the SOL, electrodes were placed 2 cm below the insertions of the *gastrocnemii* over the Achille's tendon; for the MG and the LG, the electrodes were placed over the mid belly of the muscle; and for the TA, the electrodes were positioned at 1/3 of the distance on the line between the fibula and the tip of the medial malleolus. A common reference electrode was placed in a central position on the same leg (between stimulation and recording sites). For FCR, two electrodes were positioned over the muscle belly at 1/3 of the distance from the medial epicondyle to the radial styloid. Common reference electrode was placed over the medial epycondyle of the right arm.

EMG signals were amplified with a bandwidth frequency ranging from 15 to 5 kHz (gain = 1000) then digitized on‐line (sampling frequency: 5 kHz) and stored for analysis with Tida software (Heka Elektronik, Lambrecht/Pfalz, Germany).

### Electrical stimulation

The posterior tibial nerve (PTN) and the median nerve were stimulated via a single rectangular pulse (1‐msec width) delivered by a Digitimer stimulator (model DS7, Hertfordshire, UK). For PTN stimulation, the self‐adhesive cathode (8‐mm diameter, Ag‐AgCL) was placed in the popliteal fossa and the anode (5 × 10 cm, Medicompex SA, Ecublens, Switzerland) was placed over the patella. For median nerve stimulation, two silver‐chloride surface electrodes (8‐mm diameter) were positioned in line with the nerve in the cubital fossa with the cathode 2.5 cm proximal to the anode. The better stimulation site to obtain the greatest SOL or FCR H‐reflex was first located by a hand‐held cathode ball electrode (0.5 cm diameter). Once determined, the stimulation electrode was firmly fixed to this site with straps. In order to determine the stimulation intensity needed to record H‐reflex and maximal M‐wave, the intensity of stimulation was increased from H‐reflex threshold to maximal M‐wave (M_max_). Four stimulations were recorded at each intensity. To record V waves, supramaximal intensity (1.5 × M_max_ intensity) was used.

### Magnetic stimulation

MEPs were elicited by TMS (Magstim 200; Magstim Company Ltd., Carmarthen, UK) with a figure–eight shaped coil positioned over the left motor cortex. The cortical stimulation intensities for *triceps surae* muscles were assessed during submaximal voluntary contraction of the plantar flexors (10% MVC) and at rest for the FCR. The greatest amplitude for the SOL MEP with the lowest stimulation intensity (hot spot) was determine by stimulating the M1 area of *triceps surae* muscle by starting from 1 cm posterior and lateral to the vertex of the subject's head. For the FCR this optimal site was investigated in the area corresponding to Brodmann's area 4 (Mills et al. 1992). Once the optimal site was found, a mark was placed on a bathing cap worn by subjects. The coil was then secured by using a home‐made tripod with a lockable articulated arm (Otelo Factory, T&O brand, Saint Ouen L'Aumône, France) and orientated to deliver anterior‐posterior directed stimulation to the brain.

Active motor threshold was defined as the intensity for which at least two responses could be recorded for four stimulations. Then, stimulation intensity was increased by 5% of the maximum magnetic device output, until maximal MEP could be recorded. Four stimulations were delivered at each intensity. Depending on the experiment, the TMS intensity was set to evoke 50% (MEP_50%_) or 100% (MEP_max_) of maximal MEP amplitude. MEP_max_ range stimulation intensities were 70–100 and 60–80% of maximal stimulator output for the SOL and FCR, respectively. To ensure the position of the coil, MEP_max_ amplitude was frequently checked during the experiments.

### Experimental design

The first experiment was designed to assess the optimal inter‐stimuli interval (ISI) that induced the greatest modulation of triceps surae V‐waves with conditioning TMS. During sub‐maximal plantar flexion contraction (10% MVC) the supramaximal PTN stimulation was conditioned with TMS that evoked MEP_max_ at 31 ISIs ranging from −20 to +20 msec. ISI increment was set at 1 msec from 0 to ±10 msec and 2 msec from ±10 until ±20 msec. Six measurements were performed at each ISI interspaced by 8 sec of rest. Positive ISI corresponds to PTN stimulation evoked after TMS, whereas negative ISI represents PTN stimulation evoked before TMS. Test stimulation was obtained by delivering supramaximal stimulation of the motor nerve during 10% MVC of the plantar flexor muscles. This measurement was performed six times.

The second experiment was designed to analyze the effect of conditioning TMS intensity and sub‐maximal level of force contraction on V‐wave. In this session the same experimental design as described above was used. One difference is that the ISI number tested was reduced to 6, i.e. ISI 0 and 5 positives ISIs (+4, +8, +12, +16 and +20 msec). These ISIs were chosen from results of the first experiment, demonstrating a significant conditioning effect of TMS over V‐wave only with positive ISIs. The effect of conditioning TMS intensity was analyzed by comparing conditioning TMS that evoked MEP_max_ to that evoked MEP_50%_. These stimulations were used during sub‐maximal contractions performed at 10 and 50% MVC. Each condition (level of force *vs* TMS intensity) was randomly administrated. In 3 subjects, the effect of TMS evoked at MEP_max_ intensity over V‐waves was tested during maximal voluntary contraction, with the optimal ISI found in previous conditions (i.e., the one that induced the greater V‐wave facilitation).

Six subjects performed a third session to assess the conditioning effect of TMS at rest on FCR muscle. Contrary to the *triceps surae* muscle, it is easier for the FCR to evoke both H‐reflex and MEP at rest. Once the optimal sites for median nerve and TMS stimulation were found, the supramaximal median nerve stimulation intensity was conditioned with TMS that evoked MEP_max_ with the same experimental design as described in experiment 1. Single TMS, single nerve stimulation and conditioning stimulation with the optimal ISI were tested at rest and during a sub‐maximal contraction (50% of wrist flexor MVC). The effect of the conditioning TMS intensity was also tested by comparing the TMS intensity that evokes MEP_max_ and MEP_50%_.

### Data analysis

EMG and mechanical measurements were analyzed after numerical average of the six trials, except for H_max_ and M_max_ at rest which means were calculated from four trials.

**Figure 1.**
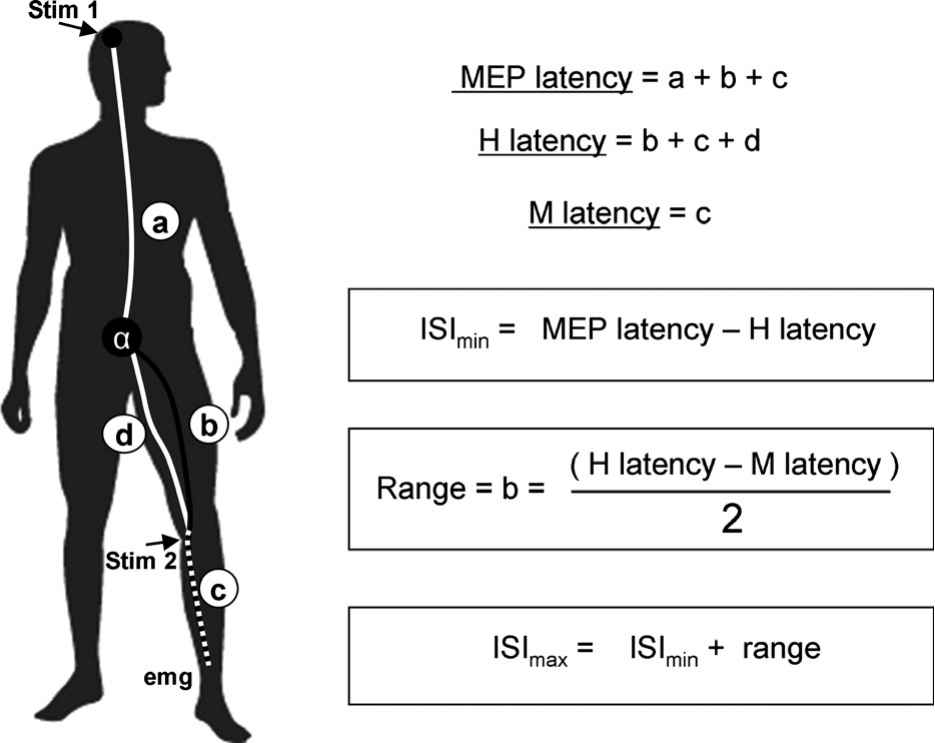
Estimation of inter‐stimuli interval (ISI) for TMS conditioning of SOL V‐waves. Arrows represent TMS (stim 1) and PTN (stim 2) stimulations sites. Each letter represent a time portion of the neural pathway. a: from stim 1 to the motoneuronal pool. b: from the spinal level to the neural stimulation site. c: from stim 2 to the EMG electrodes. d: stim 2 to the motoneuronal pool. On the right side are depicted formulas used to determine theoretical ISI from the latencies of the evoked responses (H‐reflex, M‐wave, and MEP).

EMG peak‐to‐peak amplitudes of each response (MEP_max_, H_max_, V_test_, V_cond_) were measured and normalized to the M_max_ amplitude evoked in the same condition in order to calculate the following ratios: H_max_/M_max_, MEP_max_/M_max_, V_test_/M_max_ (unconditioned response) and V_cond_/M_max_ (conditioned response) ratios. The V_cond_/V_test_ ratio was calculated to evaluate the conditioning TMS effect. MEP duration was measured from the onset of the first peak to the beginning of the silent period for SOL, MG and LG. Latencies of EMG responses were taken as the time interval from the stimulus artifact to the first peak of the responses (MEPs, V, M_max_, H_max_).These latencies were used to define theoretically the range of ISIs allowing the collision between corticospinal and antidromic volleys generated in motor axons by TMS and nerve stimulation, respectively (Fig. 1).

Peak twitch amplitudes evoked by unconditioned and conditioned PTN stimulations (Pt_test_ and Pt_cond_ , respectively) and TMS stimulations (Pt_MEP_) were analyzed over the torque plateau developed during submaximal contractions right after the stimulation. The mechanical contribution of the conditioning TMS stimulation at MEP_max_ over V‐waves (V_cond_) was calculated by subtracting Pt_test_ to Pt_cond_. This contribution was determined for each force level but only the ISI that induced the higher V‐wave facilitation was compared with Pt_MEP_ obtained for the same force level. In order to determine if motor units activated by the conditioning TMS were those involved in conditioned V‐wave, the electromechanical efficiency of the MEP_max_ (EME_MEP_) was compared with that of V_cond_ (EME_Vcond_). Because the mechanical responses evoked by PTN and TMS stimulations correspond to the activation of the triceps surae as a whole, MEP_max_, V_test_ and V_cond_ peak‐to‐peak amplitude of SOL, MG and LG muscles were summed. For each force level (10 and 50% MVC) EME_MEP_ and EME_Vcond_ were calculated as follows:
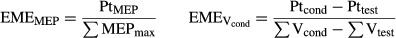


### Statistical analysis

All data are expressed by their mean ± SD. The normality of the data was tested using the Shapiro‐Wilk W test.

To assess statistical differences for M_max_, V, MEP_max_, amplitudes and latencies, V_test_/M_max,_ MEP/M_max_, a two‐way ANOVA with repeated measures was used with “muscle” (SOL, MG, LG) and “level of force” (10% MVC, 50% MVC), as within‐factors. To assess statistical effect of TMS over V‐ and M‐waves amplitudes among the 31 ISIs tested during 10% MVC, a two way ANOVA was used, with factor “muscle” and factor “ISI” (from −20 to +20 msec). One‐way ANOVA with factor “ISI” was made to analyze mechanical responses associated with each conditioned V‐wave.

For the second experiment, four‐way ANOVAs with repeated measures were used to assess statistical differences in V_cond_ amplitudes, V_cond_/M_max_ and V_cond_/V_test_ ratios with factor “ISI” (from 0 to +20 msec), factor “muscle”, factor “TMS intensity” (50% MEP_max_, 100% MEP_max_) and factor “level of force”.

With data obtained at the optimal ISI only (i.e. the ISI which showed the greatest effect of TMS conditioning on the twitch response), statistical differences for plantar flexor peak torques were assessed using a two‐way ANOVA, with factor “level of force” and factor “conditioning intensity” (no conditioning and TMS at 100% MEP_max_). FCR statistical differences for H_max_, V, MEP_max_ amplitudes and latencies, V_test_/M_max,_ MEP/M_max_, and V_cond_/V_test_ one‐way ANOVAs with repeated measures were used. The correlation between selected variables was tested with the Pearson coefficient.

When main or interaction effects were found, a post‐hoc analysis was made, using an HSD Tuckey's test. Statistical analysis was performed using STATISTICA (8.0 version, Statsoft, Tulsa, OK). Significance was accepted at *P* < 0.05.

## Results

### Unconditioned responses

For all triceps surae muscles statistical analysis revealed a significant effect of the force level on MEP_max_ and V_test_ amplitudes (*P* < 0.05) while no effect was observed for M_max_ except for the MG muscle (*P* < 0.05). As a consequence MEP_max_/M_max_ and V_test_/M_max_ ratios increased significantly when force level was enhanced from 10 to 50% of MVC (Table 1). It could be noted that these increased ratios were also observed when the force level was increased from 50% to MVC (*n* = 3).

**Table 1.** Electrophysiological data.

	Rest			
	FCR	SOL	MG	LG
M_max_ (mV)	5.44 ± 1.09	6.67 ± 0.58^§^	5.22 ± 0.84	4.93 ± 0.93^§^
H_max_ (mV)	1.53 ± 0.38	3.40 ± 0.44	1.84 ± 0.62	2.10 ± 0.83
H_max_/M_max_	0.31 ± 0.07	0.53 ± 0.06	0.32 ± 0.07	0.38 ± 0.09
MEP_max_/M_max_	0.05 ± 0.01^§^	–	–	–
H latency (msec)	18.11 ± 0.16	37.16 ± 0.97	33.71 ± 0.82	34.50 ± 1.13
M lat. (msec)	5.12 ± 0.57	10.89 ± 0.69	8.27 ± 0.32	7.25 ± 0.45
MEP latency (msec)	23.69 ± 1.01	–	–	–
	Active			
10% MVC	M_max_ (mV)	8.05 ± 1.69	3.78 ± 0.45	6.83 ± 1.11^*^
	V (mV)	0.22 ± 0.0^*^	0.28 ± 0.19^*^	0.11 ± 0.02^*^
	V_max_/M_max_	0.03 ± 0.01^**^	0.06 ± 0.03^**^	0.02 ± 0.00^**^
	MEP_max_/M_max_	0.06 ± 0.03^*^	0.04 ± 0.01^**^	0.03 ± 0.00^**^
	V lat. (msec)	36.67 ± 0.59	33.18 ± 0.71	33.73 ± 1.07
	M lat. (msec)	11.02 ± 0.46	8.86 ± 0.59	7.56 ± 0.24
	MEP lat. (msec)	42.79 ± 1.99	36.17 ± 1.24	37.88 ± 1.32
50% MVC	M_max_ (mV)	8.19 ± 1.81	4.15 ± 0.55	4.66 ± 0.73
	V (mV)	1.03 ± 0.35	0.70 ± 0.04	0.27 ± 0.06
	V_max_/M_max_	0.13 ± 0.03	0.10 ± 0.03	0.05 ± 0.00
	MEP_max_/M_max_	0.10 ± 0.02	0.09 ± 0.01	0.13 ± 0.04
	V lat. (msec)	35.08 ± 1.05	32.28 ± 0.89	31.84 ± 1.11
	M lat. (msec)	10.96 ± 0.58	8.49 ± 0.47	7.54 ± 0.52
	MEP latency (msec)	38.33 ± 1.98	37.06 ± 1.73	37.26 ± 1.27

Data are mean ± SD. SOL, Soleus; MG, Medial Gastrocnemius; LG, Lateral Gastrocnemius (n = 10); FCR, Flexor Carpi Radialis (n = 6).§significant difference with active condition for both level of force, at *P* < 0.05. Statistical differences with 50% MVC condition : *P < 0.05, **P < 0.01.

No significant effect of force level was obtained on MEP_max_, V_test_ and M_max_ latencies (Table 1). However, MEP_max_ latency was significantly longer (~5 msec) than V_test_ latency. Similar delay latency was observed at rest for the FCR muscle between MEP_max_ and H_max_. Despite the fact that H‐reflex was obtained only at rest for triceps surae muscles, this 5 msec time delay was also observed between MEP_max_ and H_max_ latencies, while H_max_ and V_test_ latencies were similar.

To be effective, the collision between corticospinal volley initiated by TMS and antidromic volley evoked by supramaximal nerve stimulation should occur between the spinal cord and the electrical stimulation site over the motor nerve (Fig. 1). The range of ISIs calculated for the SOL, according to formulas depicted in Fig. 1, was 13.13 ± 0.69, from +5.63 ±1.66 (ISI_min_) to +18.76 ±1.99 (ISI_max_). This range was slightly shorter for the MG (12.72 ± 0.35) and LG (11.71 ± 1.91) muscles starting at +7.46 ±0.76 and +7.82 ±3.35 and ending at +18.15 ±0.18 and +18.93 ±1.70, respectively. Positive values obtained for starting‐ending ISIs indicated that collision could occur only when conditioning TMS was evoked before nerve stimulation. This result was also observed for FCR muscle at rest for which TMS should be evoked from +6.14 ±0.43 to +12.72 ±0.50. In calf muscles, MEP durations were ranging from 13.37 ± 1.64 msec (MG) to 15.26 ± 2.55 msec (LG) at 50% MVC and from 14.26 ± 0.91 msec (MG) to 15.82 ± 1.77 msec (SOL) at 10% MVC. No statistical differences were found neither between force level nor muscles (*P* > 0.05). At rest, FCR MEP duration was 14.17 ± 2.08 msec.

### Conditioned responses

**Figure 2.**
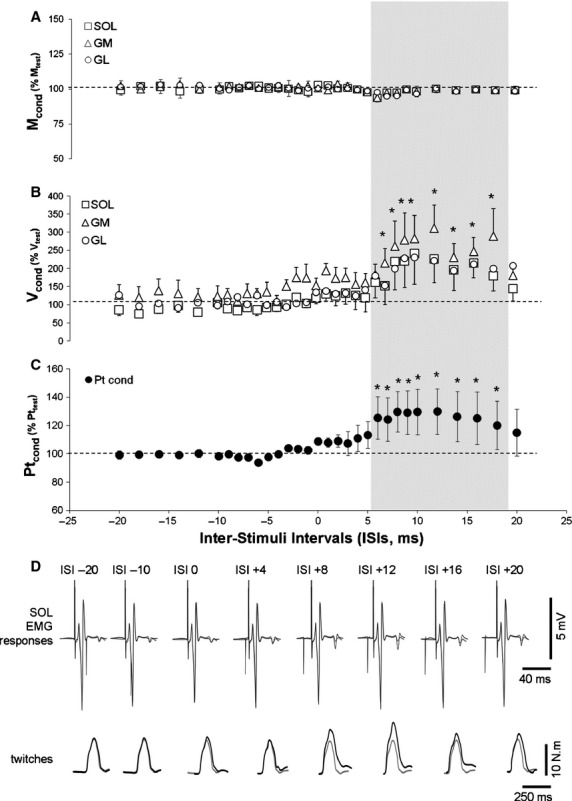
TMS induced facilitation on triceps surae muscles according to the ISI at 10% MVC. Conditioned responses are expressed in percentage of the unconditioned. Hatched zone represent the theoretical calculated ISIs. *significant difference between conditioned and unconditioned responses (*P* < 0.05). (A) conditioning effect on M waves. (B) conditioning effect on V waves. (C) effect on plantar flexion peak torque. (D) typical traces of one representative subject in EMG of SOL muscle (top traces) and in plantar flexion twitches (bottom traces) for unconditioned (gray traces) and conditioned responses (black traces).

In all experiments and whatever the testing conditions (ISI, force level or TMS intensities), no significant effect of conditioning stimulation was found on M_max_ amplitude (*P* > 0.05, Fig. 2A and D).

In experiment 1 the conditioning effect of TMS on PTN stimulation was analyzed during voluntary plantar flexion (10% MVC) from ISI ranging from −20 to +20 msec. A significant effect of conditioning TMS evoked at MEP_max_ intensity was found on V‐waves responses for all calf muscles (*P* < 0.05). V_cond_ were significantly increased compared to V_test_ for ISIs ranging from +6 to +18 msec (Fig. 2B). These ranges (~12 msec) as well as starting‐ending values of ISIs were close to those determined previously by using MEP_max_, H_max_ and M_max_ latencies. The maximal increase of V_cond_ compared to V_test_ was of 227.7 ± 109.9%, 332.5 ± 95.2% and 230.51 ± 68.30% and was obtained at ISIs of +13 ±2.32 msec, +12 ±1.70 msec and +14 ±1.46 msec for Sol, MG and LG, respectively. The TMS conditioning effect had also a significant impact on the superimposed twitch. Pt_cond_ was significantly higher than Pt_test_ for ISIs ranging from +6 to +18 msec with maximum facilitation effect (129.98 ± 16.09%) obtained at an ISIs of 13.3 ± 1.28 msec (Fig. 2C).

**Figure 3.**
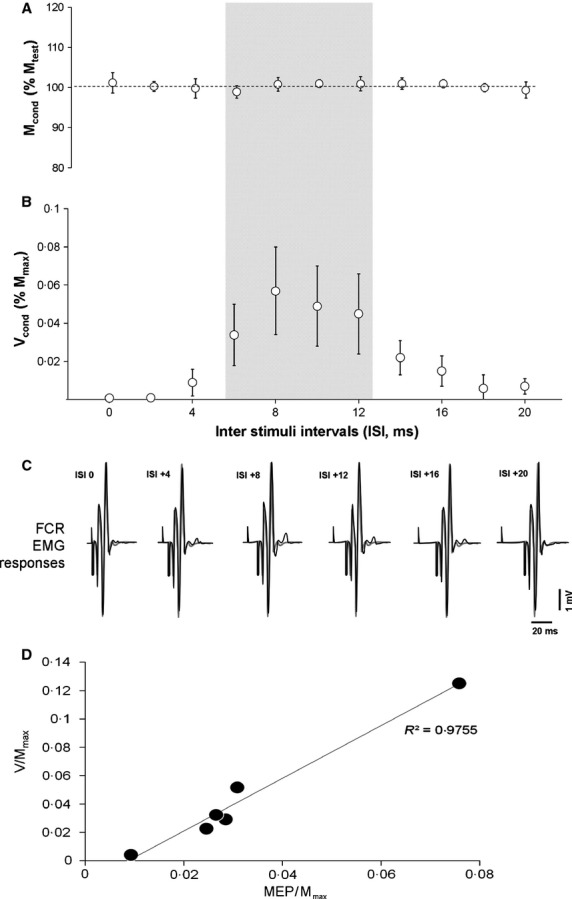
TMS induced facilitation at rest on FCR muscles according to the ISI. Conditionned responses are expressed in percentage of the unconditioned. Hatched zone represent the theoretical calculated ISIs. *significant difference between conditioned and unconditioned responses (*P* < 0.05). (A) conditioning effect on M waves. (B) conditioning effect on V waves. (C) typical traces of one representative subject for unconditioned (gray traces) and conditioned responses (black traces). (D) Relationship between FCR MEP and V waves obtained at rest (*y* = 1.86*x* − 0.01).

Results obtained at rest in FCR muscle demonstrated that, while no F‐wave was observed for unconditioned condition, a later wave was elicited with conditioning TMS (Fig. 3). The latency of this wave (17.98 ± 0.56 msec) was similar to the H‐reflex latency (18.11 ± 0.16 msec). The maximal response observed (5.7 ± 2.3% of M_max_) was evoked for mean ISI of +8.25 ±1.11 msec. The normalized amplitude of this response was not significantly different than the MEP_max_/M_max_ ratio, and a significant linear relation was obtained between these data (Fig. 3D). For FCR recordings, no significant effects of voluntary activation (50% MVC VS rest) and conditioning intensity were found regarding optimal ISIs. FCR muscle depicted same results during 50% MVC as in calf muscles, the ratio V/Mmax being increased by 135.18 ± 33.36% and by 167.18 ± 16.26%, respectively with 50% MEP_max_ and 100% MEP_max_ conditioning TMS. This last result allows a reasonable comparison between upper and lower limb muscles tested in the present study.

**Figure 4.**
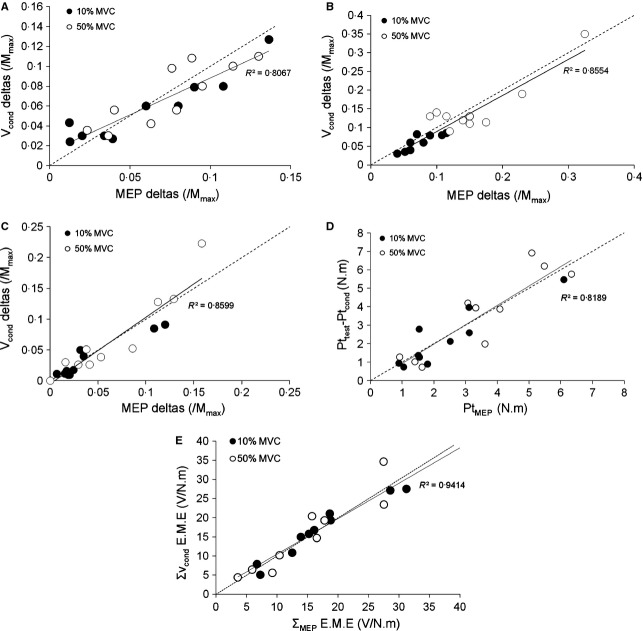
Relationships between electrophysiological and mechanical data associated with V‐wave and cortical stimulation. Dotted line represents the identity line (*y* = *x*). Relationships between V_cond_ deltas and MEP deltas are depicted for SOL (A, *y* = 0.74*x* + 0.01), GM (B, *y* = 0.96*x *− 0.01) and GL (C, *y* = 1.08*x *− 0.004) muscles. Mechanical contribution of TMS on plantar flexion twitches is plotted against mechanical responses of TMS only (D, *y* = 1.05*x *− 0.01). Electromechanical efficiency (EME) of triceps surae muscles associated with TMS is plotted against EME associated with conditioned V‐waves (E, *y* = 0.93*x* + 1.21).

In experiment 2 the effect of conditioning TMS intensity (MEP_max_ and MEP_50%_) and force level (10% and 50% MVC) over V‐wave has been investigated. In overall, and considering the ISI which induced the greatest facilitation, increase the level of force or decrease the conditioning TMS intensity induced a decrease in V_cond_/V_test_ ratio of the triceps surae muscles. This ratio decreased from 3.28 ± 1.10, 5.49 ± 1.73 and 4.26 ± 1.41 to 1.81 ± 0.22, 4.45 ± 1.48 and 2.46 ± 0.54 at 10% MVC when conditioning intensity decrease from MEP_max_ to MEP_50%_ for SOL, MG and LG muscles, respectively. A similar decrease was observed when the force level was at 50% MVC (decrease from 2.54 ± 0.33, 3.05 ± 0.78 and 3.24 ± 0.71 to 1.66 ± 0.27, 2.12 ± 0.48 and 2.32 ± 0.43 for SOL, MG and LG muscles, respectively). The effect of conditioning stimulation intensity on V‐wave response was also analyzed by comparing delta MEP amplitude (ΔMEP = (MEP_max_ − MEP_50%_) / M_max_) to delta V_cond_ amplitude (ΔV_cond_ = (V_cond_ at MEP_max_ − V_cond_ at MEP_50%_) / M_max_) for both force levels. This analysis revealed a linear relation between both parameters for each triceps surae muscle, with slope value near 1 indicating that increases in MEP amplitude induced similar increases in V_cond_ amplitude (Fig. 4).

A significant effect of the conditioning stimulation set at MEP_max_ was found on mechanical responses. The relative increase of Pt_cond_ compared to Pt_test_ was of 29.82 ± 15.09% and 17.63 ± 5.31% for 10 and 50% MVC, respectively. For both force levels no significant difference was observed between Pt_MEP_ and the relative mechanical contribution of the conditioning stimulation determined by subtracting Pt_test_ to Pt_cond_. Moreover, a linear relation was obtained between these two parameters (Fig. 4D).

Finally, the statistical analysis demonstrated a linear relation between electromechanical efficiency of MEP_max_ and V_cond_ (see Material and Methods) with a slope close to the identity line indicating that, whatever the force level, the motor units activated by V_cond_ have the same EME than those activated by MEP_max_ (Fig. 4E).

## Discussion

The main finding of the study was an increase of electrophysiological and mechanical responses related to the V‐wave for all calf muscles when the supramaximal electrical nerve stimulation was conditioned by TMS during weak voluntary contractions. The amplitude of mechanical and electrical responses associated with the conditioned V‐wave was correlated with single cortical stimulation. This original result suggests that V‐wave response can be a reliable clue of the amount of cortical neural drive addressed to the motorneuronal pool.

### Inter‐stimuli interval

For all calf muscles, significant V_cond_ increases in comparison to V_test_ were obtained for ISIs ranging from +6 to +18 msec with a maximal facilitation occurring for optimal ISIs of ~+13 msec. The total range of effective ISIs can be related to the temporal dispersion of MEP responses, which is larger than V‐wave response. In fact, duration of MEP responses, whatever the muscle and condition was closed to the range of ISIs that induced a significant facilitation of V‐wave amplitude. Moreover, these facilitation effects had also a significant impact on the superimposed twitch amplitude evoked during weak voluntary contraction. Similar results were obtained when conditioning TMS intensity was modulated from MEP_max_ to MEP_50%_ or when the level of submaximal voluntary contraction was increased from 10 to 50% MVC. Ranges as well as starting‐ending values of ISIs that induced these increases of evoked responses were close to those determined by using MEP_max_, H_max_ and M_max_ latencies. These latter define ISIs that allow an effective collision between efferent and antidromic impulses generated, respectively, by TMS and nerve stimulation occurring at the neural pathway travelled by the two impulses, i.e. on motor axons between the spinal cord and the stimulation site over the motor nerve. These results suggest that the V‐wave facilitation induced by conditioning TMS could be linked to the removal of efferent impulses by collision with anditromic impulses (Upton et al. 1971).

However, conditioning stimulations had been evoked during submaximal voluntary contractions that could affect the effect of TMS on V‐waves amplitude leading to misinterpretation of the nature of the collision. Therefore, to assess whether this effect was related to the background EMG activity linked to weak voluntary contractions, additional data obtained at rest were recorded in six subjects on FCR muscle. The results showed that conditioning TMS effect over supramaximal nerve stimulation can induce, after the M_max_ response, a late electrophysiological response with a similar latency to that of the H‐reflex. As for the calf muscles, the range of ISIs as well as starting‐ending values that induced this response were close to those determined by using MEP_max_, H_max_ and M_max_ latencies. These results suggest that neural processes generating the late electrophysiological response at rest were similar to those involved in the facilitation of the V‐wave during weak voluntary contraction.

Several possible neural mechanisms can explain the late muscle response occurrence at rest. The first possibility, due to facilitation effect of the cortical descending impulses generated by TMS on the motoneuronal pool, is an increase of the F‐wave. This response recorded at rest results from antidromic activation of a number of motoneurones following supramaximal nerve stimulation (Thorne 1965; Panayiotopoulos and Chroni 1996). The F‐wave is known to present low persistence (proportion of stimulation in which an F‐wave is present) and high variability in the latency (Panayiotopoulos and Chroni 1996). In the present study, the results obtained for the FCR muscle demonstrating a high persistence (late response present for every trail) and low variability in the latency (17.98 ± 0.56 msec, variation coefficient <3%) allow to exclude a facilitation effect of the cortical descending impulses on motoneurones as a possible neural mechanism involved in the late muscle response. This is reinforced by the fact that the test condition (supramaximal nerve stimulation evoked alone) never elicited an F‐wave response.

The second neural mechanism that can be evoked is that the late muscle response corresponds to the activation by afferent volleys of a number of motorneurones for which the collision between efferent and antidromic impulses generated by TMS and nerve stimulation, respectively, had taken place. This is in accordance with the results obtained in our study, showing that, for ISI that induces maximal facilitation, the conditioning MEP_max_ and late response amplitudes were linearly correlated with similar mean amplitude values (5.0 ± 1.0 vs. 5.7 ± 2.3% for late response and MEP_max_ ,respectively). These results indicate that MU involved in the late muscle response is that which is activated by the cortico‐spinal impulses initiated by TMS. The neural mechanisms that are responsible for this response obtained at rest are likely the same than those involved in the V‐wave facilitation obtained during weak voluntary contractions.

### Conditioning intensity and force level effect

Despite the fact that V‐wave amplitude can be affected by neural mechanisms, including changes in motoneuron responsiveness and synaptic transmission efficacy between Ia afferent and Mnα (e.g., pre‐ and postsynaptic mechanisms) (Pierrot‐Deseilligny and Burke 2005; Carroll et al. 2011), its modifications have been extensively used as a measurement reflecting the level of efferent neural drive addressed to spinal Mnα during MVC (Aagaard et al. 2002; Pensini and Martin 2004; Duclay and Martin 2005; El Bouse et al. 2013). In fact, motoneurons excitability can be modulated by many others inputs than cortical projections. For instance, it is well known that spinal excitability can be modulated differently according to the initial state of the muscle, i.e. in static position or during lengthening, because of peripheral inputs from Ia discharge (Pinniger et al. 2001). Moreover, such a TMS‐conditioning maneuver, as used in the present study, may be influenced by afferent feedback from muscle lengthening (Grosprêtre et al. 2014). In the present study, modulation of V‐wave amplitude was tested only during isometric contraction, without varying peripheral input onto motoneuronal pool. Therefore, we cannot confirm that V‐wave modulation is exclusively under cortical influence. Nevertheless, previous findings showed that V‐wave was not modulated according to muscle contraction mode contrary to H‐reflex amplitude, depressed during eccentric contraction (Duclay and Martin 2005). This latter result showed the relative consistence of V‐wave amplitude according to the contraction modes, despite the fact that spinal excitability can be widely modulated, showing that V‐wave has a major cortical influence.

In the present study, a modification of the level of efferent neural drive has been obtained by modulating the conditioning TMS intensity from MEP_max_ to MEP_50%_. For all calf muscles, this modulation induced a significant decrease of the V_cond_/V_test_ ratio when the force level was set at 10% MVC. Although increasing the level of force from 10 to 50% MVC‐ induced significant increase of both V_test_ and MEP_max_ amplitudes, similar V_cond_/V_test_ ratio modulations have been observed when the conditioning intensity was decreased. Similar results observed in three subjects during maximal voluntary contraction, thus providing greater amplitudes of V‐ and MEP responses, indicate that this TMS conditioning effect could be observed at higher levels of force. Moreover, for all calf muscles and whatever the force level (10 and 50% MVC), linear relations with slopes close to 1 have been obtained between ΔMEP and ΔV_cond_ (see Results). These results could indicate that a decrease in the cortical neural drive addressed to spinal Mn decrease the efferent motor output limiting the anditromic collision phenomenon and the portion of evoked afferent volley that could reach the muscle. Consequently, it can be assumed that the V‐wave amplitude modulations can reflect changes in the cortical neural drive addressed to spinal Mnα, this latter defining their recruitment and/or discharge rate. However, it was rightly suggested by Matthews (1986) that preceding activity may influence the amplitude of the recorded reflexes, given the fact that amplitude of the EMG responses naturally increase as the background EMG activity increase. This “automatic gain compensation” may affect the responsiveness of the motoneuronal pool to the cortical stimulation. Nevertheless, in the present study when we used several TMS intensities during the same force level, meaning that motoneurons background activity is constant (same amount of voluntary neural drive and peripheral feedback from muscle contraction), we also found that the amplitude of facilitated V‐wave depends on TMS intensity used, thus on cortical output. Moreover, the experiment carried out at rest on FCR muscle also provides clues that V‐wave can results from cortical output. In fact, at rest and by carefully controlling subject's arm position, we can ensure that peripheral output is not sufficient to modulate motoneurons activity, thus “minimizing the risk of automatic gain compensation”.

The effects of conditioning TMS on the V‐wave have also induced a significant increase of the superimposed twitches size recorded at 10 and 50% of the MVC. The mechanical contribution of the conditioning TMS at an intensity of MEP_max_ was obtained by subtracting, at both force levels, unconditioned to conditioned superimposed twitches. This contribution appeared to be linearly correlated with the twitch evoked by MEP_max_, reinforcing the fact that V‐wave amplitude may reflect the amount of cortical output. A possible explanation is that the same MUs could be involved in V‐wave and in MEP responses. Indeed, considering the triceps surae as the whole, it is demonstrated that the electromechanical efficiency of the MUs activated by the MEP_max_ and V_cond_ are linearly correlated with a slope close to the identity line, showing that same MUs could be activated in both responses.

To conclude, this study demonstrates, by using an original conditioning maneuver,that the V‐wave response consists of a volley of afferent impulses that are allowed to reach the muscle because of the removal of antidromic impulses by collision with efferent impulses generated by TMS and further confirm initial conclusions of Upton et al. (1971). The main explanation lies in the participation of the same motor units in V_cond_ and in MEP_max_ amplitude, suggesting that V‐wave response can be an index of the cortical neural drive addressed to spinal Mnα. However, despite a large cortical influence, further experiments are needed to assess whether V‐wave could also be subjected to peripheral influences such as somatosensory feedbacks.

Because V‐wave recording is easy to implement and not time‐consuming, this method appears to be helpful associated with other measurements (e.g. level of activation, H‐reflex) in studies analyzing the potential mechanisms mediating neural adjustments following acute or chronic physical exercise. For instance, this is important especially for fatiguing exercise for which measurements of neuromuscular function must be performed as soon as possible after exercise ending.

## Conflict of Interest

No conflicts of interest, financial or otherwise, are declared by the author(s).
